# Relevant Characteristics Analysis Using Natural Language Processing and Machine Learning Based on Phenotypes and T-Cell Subsets in Systemic Lupus Erythematosus Patients With Anxiety

**DOI:** 10.3389/fpsyt.2021.793505

**Published:** 2021-12-10

**Authors:** Xi-xi Gu, Yi Jin, Ting Fu, Xiao-ming Zhang, Teng Li, Ying Yang, Rong Li, Wei Zhou, Jia-xin Guo, Rui Zhao, Jing-jing Li, Chen Dong, Zhi-feng Gu

**Affiliations:** ^1^Department of Rheumatology, Affiliated Hospital of Nantong University, Nantong University, Nantong, China; ^2^Joint Research Center, Affiliated Hospital of Nantong University, Nantong, China; ^3^Institut Pasteur of Shanghai, Chinese Academy of Sciences, Shanghai, China; ^4^Research Center of Clinical Medicine, Affiliated Hospital of Nantong University, Nantong, China

**Keywords:** systemic lupus erythematosus, anxiety, T-cell subsets, phenotypes, natural language processing

## Abstract

Anxiety is frequently observed in patients with systemic lupus erythematosus (SLE) and the immune system could act as a trigger for anxiety. To recognize abnormal T-cell and B-cell subsets for SLE patients with anxiety, in this study, patient disease phenotypes data from electronic lupus symptom records were extracted by using natural language processing. The Hospital Anxiety and Depression Scale (HADS) was used to distinguish patients, and 107 patients were selected to meet research requirements. Then, peripheral blood was collected from two patient groups for multicolor flow cytometry experiments. The characteristics of 75 T-cell and 15 B-cell subsets were investigated between SLE patients with- (*n* = 23) and without-anxiety (*n* = 84) groups by four machine learning methods. The findings showed 13 T-cell subsets were significantly different between the two groups. Furthermore, BMI, fatigue, depression, unstable emotions, CD27^+^CD28^+^ Th/Treg, CD27^−^CD28^−^ Th/Treg, CD45RA^−^CD27^−^ Th, and CD45RA^+^HLADR^+^ Th cells may be important characteristics between SLE patients with- and without-anxiety groups. The findings not only point out the difference of T-cell subsets in SLE patients with or without anxiety, but also imply that T cells might play the important role in patients with anxiety disorder.

## Introduction

Systemic lupus erythematosus (SLE) is the most common complex autoimmune disease characterized by chaos in the immune system, dysfunctions, and disordered proportions of immune cells (1). Patients with autoimmune diseases appear to be associated with increased risks of psychotic disorders (2). The epidemiologic study has revealed striking links between several autoimmune diseases and psychosis (3). Compared with other autoimmune diseases, such as sicca syndrome (PSS), SLE patients are more likely to suffer from anxiety disorders (12 vs. 4%) (4). Anxiety appears as a general and major

stress disorder for SLE patients, resulting in a worse prognosis, more serious mental diseases, and even suicide (5).

Increasing evidence shows that SLE patients with anxiety may be a cognitive dissonance caused by abnormal activations of immune systems (6), and can be characterized by high levels of pro-inflammatory cytokines (7), such as TNF, IFN-γ, IL-10, IL-6 (8, 9). Some cytokines are not only increased significantly in the serum of anxious patients (10, 11) but also in SLE patients with anxiety (12, 13). However, cytokines are produced by a variety of cells including immune and many other types of cells, such as T cells, B cells, endothelial cells, etc. (14, 15), which may not directly reflect the relationships between the disordered proportions of immune cells and mental illness. Research in psychoneuroimmunology has demonstrated that the status of immune system, especially the proportion of immune cells, could influence psychological stress (16), and the immune-brain interactions play vital roles in the initiation and development of psychiatric disorders (17). An increase of CD4^+^ T-cells and damage of the amygdala are found in anxious mice (14), and the decrease of CD3^+^ T-cells may indicate an improvement of cognition in SLE mice (13). Thus, the investigation for the associations between subsets of T-cell and anxiety in SLE patients could give rise to the study of the mechanism of SLE patients with psychotic disorders and ongoing inflammatory processes. Nevertheless, we also did a conventional analysis of the B-cell subsets.

In this study, we surveyed the distribution of 75 subpopulations of T-cell and 15 subpopulations of B-cell from 107 SLE patients using flow cytometry and investigated their differences between patients with- (*n* = 23) and without anxiety (*n* = 84). Moreover, machine learning methods were used to establish models combining clinical information, laboratory indicators, and disease phenotypes for further selecting important characteristics of SLE patients with anxiety (SLE-A group) and SLE patients without anxiety disorders (SLE-NA group). Our results demonstrated that several characteristics including BMI, fatigue, depression, unstable emotions, and CD27^+^CD28^+^ Th/Treg, CD27^−^CD28^−^ Th/Treg, and other characteristics which can be used as objective indexes for judging SLE patients with anxiety and make up for the deviation of subjective consciousness in scales survey.

## Patients and Methods

**Figure 1:**
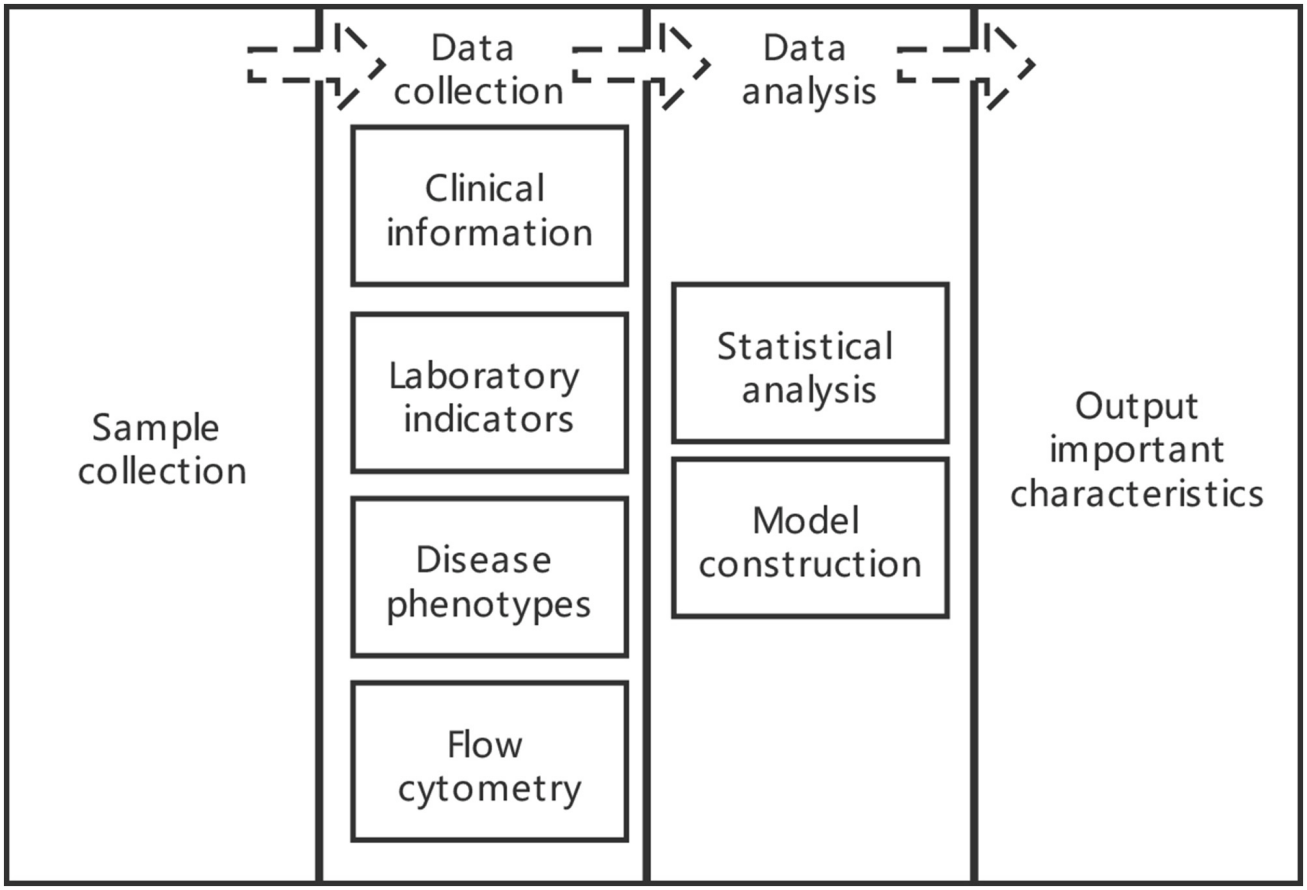
Study design and analysis plan flow diagram.

Study design and analysis plan flow diagram was shown in Figure 1.

### Sample Collection

All procedures in this trial, including sample collection, processing, freezing, laboratory analysis, etc., were performed according to the principles of laboratory practice with established standard operating procedures and protocols in the research center of clinical medicine at the Affiliated Hospital of Nantong University. The study, including the human tissue collection, was approved by the Ethics Committee of the Affiliated Hospital of Nantong University (2017-K003), and written informed consent was obtained from all of the participants, according to the Declaration of Helsinki. The study was conducted during 2019–2020.

In total, 107 SLE patients, which met the diagnostic criteria of the American Society of Rheumatology (v1997, v2012), were enrolled in this study from the Affiliated Hospital of Nantong University, China. The exclusion criteria included: other autoimmune diseases and active infection (including hepatitis B or C virus, Epstein-Barr virus, human immunodeficiency virus, or Mycobacterium tuberculosis infection); patients suffering from other autoimmune diseases and other severe mood disorders; family history of genetic diseases; cognitive impairment or inability to understand the researchers' words; major personal or family events in the past 2 months. The hospital Anxiety and Depression Scale (HADS) was used to assess the mental status of the patients (anxiety status was diagnosed by the HADS scores≥ 8).

### Data Collection

#### Clinical Information

The clinical information, such as age, gender, body mass index (BMI), course, etc. was collected by asking patients questions. Compared with the Self-Rating Anxiety/Depression Scale and Beck Depression Inventory (BDI) etc., HADS has the advantages of lower time cost and higher accuracy in assessing rheumatic mental illness (18, 19) and has better reliability and validity in SLE patients with anxiety (20). It was used to assess the mental status of the patients (depression status was diagnosed by the HADS scores ≥ 8). The Pittsburgh Sleep Quality Index (PSQI) was employed to evaluate the quality of SLE patients (more than 5 points was assigned the status of sleep disorder). The Multidimensional Fatigue Inventory Scale (MFI-20) was employed to evaluate the fatigue of SLE patients (the higher the score, the more tired the patient felt). The Systemic Lupus Erythematosus Disease Activity Index (SLEDAI) was employed to evaluate the activity of SLE (more than 4 points were assigned active status of disease). All clinical information was deposited in Table 1.

**Table 1:** Clinical characteristics of SLE patients.

**Clinical characteristics**	**SLE-A**	**SLE-NA**	***P*-value**
	**(*n* = 23)**	**(*n* = 84)**	
Age^b^	40.96 ± 13.79	38.24 ± 13.57	0.398
Gender: female (%)^c^	20.00 (87.00)	79.00 (94.00)	0.485
BMI kg/m^2^^c^			**0.003**
<18.5	2.00 (8.70)	7.00 (8.50)	
18.5–23.9	8.00 (34.80)	58.00 (70.70)	
>24	13.00 (56.50)	17.00 (20.70)	
Place of residence^c^			0.905
City	11.00 (47.80)	39.00 (46.40)	
Others	12.00 (52.20)	45.00 (53.60)	
Marital status^c^			0.992
Married	17.00 (73.90)	62.00 (73.80)	
Others	6.00 (26.10)	22.00 (26.20)	
Education^c^			>0.999
≤ Compulsory education	19.00 (82.60)	69.00 (82.10)	
>Compulsory education	4.00 (17.40)	15.00 (17.90)	
Income (yuan/year)^c^			**0.046**
<15,000	5.00 (21.70)	42 (50.60)	
15,000–33,000	9.00 (39.10)	22 (26.50)	
>33,000	9.00 (39.10)	19 (22.90)	
Job: yes (%)^c^	18.00 (78.30)	56 (66.70)	0.286
Birth history: yes (%)^c^	19.00 (86.40)	59 (71.10)	0.145
Depression: yes (%)^c^	13.00 (56.50)	7.00 (8.30)	**<0.001**
Sleep disorder: yes (%)^c^	11.00 (50)	21.00 (25.00)	**0.023**
Fatigue^b^	61.00 ± 13.20	47.02 ± 13.07	**<0.001**
SLEDAI scores^a^	6.00 (3.00, 12.00)	5.50 (4.00, 10.00)	0.816

a *Values are presented as the median (25th and 75th percentiles) and analyzed by Mann-Whitney U-test*.

b *Values are presented as the mean ± SD and analyzed by independent samples T-test*.

c *Values are presented as the number (%) analyzed by chi-square tests*.

*The P-value is preserved by three decimal places, and the rest is preserved by two decimal places, and P-values <0.05 are bold*.

*SLE-A, systemic lupus erythematosus patients with anxiety disorders; SLE-NA, systemic lupus erythematosus patients without anxiety disorders; BMI, body mass index; SLEDAI, systemic lupus erythematosus disease activity index*.

#### Laboratory Indicators

Laboratory indicators including routine blood tests, liver and renal functions biochemical examination, and complement 3/4, C-reactive protein (CRP), erythrocyte sedimentation rate (ESR), etc. were recorded. Since not all patient examination items were the same, after consulting a specialist, we deleted most of the missing indicators and unimportant indicators for patients. All laboratory indicators were deposited in Supplementary Table 1.

#### Disease Phenotypes

We sorted out the electronic records whose content referred to the part of the SLE Symptom Checklist (SCC) (21) that recorded the main phenotypes of 107 patients, and removed part of the obvious noise that interfered with the word segmentation statistics. Among them, three patients had no records. The R package named jiebaR, an efficient R language Chinese word segmentation package, was used to segment the electronic phenotype records of the 107 patients. The main phenotypes of SLE patients were included in a custom dictionary. We extracted the keywords whose parts of speech were nouns and adjectives in each group of each electronic record, counted the frequency of the two groups of keywords, and did a statistical difference analysis. All disease phenotypes were deposited in Table 2.

**Table 2:** Disease phenotypes characteristics of SLE patients.

**Disease phenotypes**	**SLE-A**	**SLE-NA**	***P*-value**
	**(*n* = 23)**	**(*n* = 84)**	
Weak: yes (%)	18.00 (78.30)	35.00 (43.20)	**0.003**
Uncomfortable in the eyes: yes (%)	13.00 (56.50)	26.00 (32.10)	**0.033**
Less appetite: yes (%)	9.00 (39.10)	15.00 (18.50)	**0.038**
Unstable emotions: yes (%)	16.00 (69.60)	27.00 (33.30)	**0.002**
Pain in muscles: yes (%)	9.00 (39.10)	22.00 (27.20)	0.268
Skin rash: yes (%)	10.00 (43.50)	25.00 (30.90)	0.259
Itch: yes (%)	12.00 (52.20)	32.00 (39.50)	0.278
Pimples: yes (%)	6.00 (26.10)	13.00 (16.00)	0.427
Hypertrichosis: yes (%)	2.00 (8.70)	17.00 (21.30)	0.288
Giddiness: yes (%)	11.00 (47.80)	26.00 (32.10)	0.164
Loss of concentration: yes (%)	10.00 (43.50)	21.00 (25.90)	0.104
Spontaneous bruises: yes (%)	10.00 (43.50)	22.00 (27.20)	0.135
Disturbed memory: yes (%)	13.00 (56.50)	31.00 (38.30)	0.118
Weight gain: yes (%)	4.00 (17.40)	26.00 (32.10)	0.169

*Values are presented as the number (%) analyzed by chi-square tests*.

*The P-value is preserved by three decimal places, and the rest is preserved by two decimal places, and P-values <0.05 are bold*.

#### Flow Cytometry

For each SLE patient, 5–10 ml peripheral blood was collected using heparin sodium anticoagulant tube and transported to the laboratory under 4°C. Peripheral blood mononuclear cells (PBMCs) were collected by Lymphoprep (Axis-Shield) density gradient centrifugation. Antibody information for types of immune cells used in the selection process of flow cytometry (FACS Fortessa from American BD company) is shown in Supplementary Table 4. Red blood cells were lysed with Red Blood Cell Lysis Buffer (American BD company). Fixation Buffer (American Biolegend company) was added to fix PBMCs and using Macs Buffer (1xPBS add 1%FBS, 2.5 ml EDTA come from American GIBCO company) washed out the excess reagent during the whole experiment. FlowJo V9 was used for data analysis. All information was deposited in Supplementary Tables 2, 3.

### Data Analysis

#### Statistical Analysis

All analyses were performed using R language if not specially declared. The differences between SLE-A and SLE-NA groups were elevated by an independent sample of *T*-test when the samples of different groups were from the same normal distribution (with the same variance), otherwise, the Mann-Whitney U test was used in the cases. The level of *P* < 0.05 was used to evaluate the statistical significance.

#### Model Construction

We matched clinical information, laboratory indicators, disease phenotypes, and cell subpopulation data separately to construct five types of original data sets. Then, the use of the original data sets was compared and the oversampling and the algorithm of Syntic priority oversampling technology (SMOTE) methods (22) which was used to make up for the imbalance in the number of cases included in the data set on various models, including Lasso regression (LR) (23), Random Forest (RF) (22) and XGBoost (24). When applying the oversampling and SMOTE method, the SLE-NA group to 84 cases (+10 cases) and the SLE-A group to 70 cases (±10 cases) were always controlled in proportion. Each data set was subjected to five-fold cross-validation, and finally the average of the five areas under receiver operating characteristic curves (AUC) values were used as the final judgment criterion to initially screen out the best combined data set, sampling method, and machine learning model. After that, the data set was rebalanced by the best sampling methods, then divided into training and testing data by a ratio of seven to three, and the final model was rebuilt. According to the characteristics weight ranking, the top 25 characteristics were selected. In processing data, we replaced the vacancy value of each data set with the median value. The main R packages are shown in Supplementary Table 5.

## Results

### SLE Patients With Anxiety Had Higher BMIs and Associated Symptoms of Depression and Fatigue as Well as Unstable Emotions and Weak

In this study, 107 SLE patients were recruited during 2019–2020, 23 of which were coupled with anxiety disorders (SLE-A group). The incidence rate of anxiety in SLE patients showed slightly higher than other diseases (21 vs. 4%, Table 1), which was consistent with the previous study (4). Interestingly, no statistically significant differences were observed between SLE-A and SLE-NA (84 SLE patients without anxiety) groups in age, gender, education level, marital status, place of residence, SLEDAI score, and other laboratory indicators (see Supplementary Table 1). BMI, income, sleep disorders, depression, and fatigue were significantly different between the two groups (see Table 1). Among our SLE patients, the BMI of the SLE-A group was higher than that of the SLE-NA group (*P* < 0.05), the income was higher than that of the SLE-NA group, and they were more prone to sleep disorders and psychological problems such as depression and fatigue. There had disease phenotypes that were also signs of psychological problems. For example, the SLE-A group would feel more weak, feel uncomfortable in the eyes, have less appetite, and present unstable emotions (see Table 2), which might mean that SLE patients with anxiety may exist unique external manifestations.

### Dramatical Alteration of γδ2T and 12 Subsets of Th and Treg Cell Subsets in SLE-A Patients

Previous studies demonstrated that the dysregulated immune response and an abnormal subpopulation of immune cells play a critical role in the mental complications of SLE patients. We wondered whether the subsets of T cells were involved in the anxiety disorder for SLE patients. The populations of major types of T cells, such as αβT and γδ1T cells, showed no statistically significant differences between the two groups, except γδ2T (see Supplementary Table 3 and Table 3). Furthermore, we investigated the subpopulations of main types of T cells and found that the detectable subsets of CD8^+^ T-cells appeared very few differences between the SLE-A and SLE-NA groups (see Supplementary Table 3). Notably, the abundance of CD27^+^CD28^+^ and CD45RA^+^HLADR^+^ Th cells in the SLE-A group was significantly lower than that in the SLE-NA group, while the proportion of CD27^−^CD28^−^, CD45RA^−^HLADR^−^, and CD45RA^−^CD27^−^ Th cells showed a significant increasing trend. In addition, we observed that the proportion of CD27^+^CD28^+^ Treg cells in the SLE-A group was significantly reduced compared with the SLE-NA group. At the same time, a significant increase was noticed in the four subgroups of Treg cells in the SLE-A group, CD27^−^CD28^−^, CD45RA^−^HLADR^−^, CD45RA^−^CD27^−^, and PD1^−^CD28^−^ Treg cells. Interestingly, the CD27^+^CD28^+^ Th/Treg subpopulations of the SLE-A group were significantly reduced, while the abundance of CD27^−^CD28^−^ Th/Treg cells was greatly increased (see Table 3). We also investigated the subpopulations of the main types of B cells and found no differences between the two groups (see Supplementary Table 2).

**Table 3:** The abundance of 13 subsets of immune cells in SLE-A and SLE-NA groups.

**T-cell subsets**	**SLE-A**	**SLE-NA**	***P*-value**
	**(*n* = 23)**	**(*n* = 84)**	
γδ2T^a^	1.40 (0.98, 2.76)	0.91 (0.33, 1.86)	**0.029**
CD27^−^CD28^+^ Th^a^	13.90 (9.75, 19.60)	9.93 (6.45, 14.30)	**0.026**
CD27^+^CD28^+^ Th^a^	70.60 (59.80, 80.30)	84.30 (74.80, 90.48)	**0.001**
CD27^−^CD28^−^ Th^a^	15.40 (0.36, 23.60)	3.67 (0.48, 10.70)	**0.039**
CD45RA^−^CD27^−^ Th^b^	28.77 ± 12.45	18.70 ± 14.30	**0.003**
CD45RA^+^HLADR^+^ Th^a^	0.35 (0.11, 16.00)	11.25 (0.29, 34.50)	**0.034**
CD45RA^−^HLADR^−^ Th^a^	50.20 (4.62, 65.60)	6.35 (1.21, 55.38)	**0.035**
CD27^−^CD28^+^ Treg^a^	8.40 (4.02, 12.80)	4.69 (2.47, 8.78)	**0.044**
CD27^+^CD28^+^ Treg^a^	84.50 (78.30, 92.00)	93.10 (86.38, 96.78)	**0.005**
CD27^−^CD28^−^ Treg^a^	3.54 (0.00, 9.64)	0.45 (0.00, 2.40)	**0.032**
PD1^−^CD28^−^ Treg^a^	2.85 (0.00, 8.21)	0.00 (0.00, 1.69)	**0.012**
CD45RA^−^CD27^−^ Treg^a^	12.80 (7.61, 17.90)	7.01 (3.17, 13.25)	**0.009**
CD45RA^−^HLADR^−^ Treg^a^	42.60 (9.26, 58.70)	11.85 (1.19, 55.63)	**0.032**

a *Values are presented as the median (25th and 75th percentiles) and analyzed by Mann-Whitney U-test*.

b *Values are presented as the mean ± SD and analyzed by independent samples T-test*.

*The P-value is preserved by three decimal places, and the rest is preserved by two decimal places, and P-values <0.05 are bold*.

*SLE-A, SLE patients with anxiety; SLE-NA, SLE patients without anxiety; Th, helper T cells; Treg, regulatory T cells*.

### BMI, Fatigue, Depression, Unstable Emotions, CD27^+^CD28^+^ Th/Treg, CD27^–^CD28^–^ Th/Treg, CD45RA^–^CD27^–^ Th, and CD45RA^+^HLADR^+^ Th Cells May Be Important Characteristics of SLE Patients With Anxiety

Based on these observations, 13 T cell subsets were significantly different between the SLE-A and SLE-NA groups, and we further explored their ability to predict SLE patients with anxiety. Clinical information, laboratory indicators, and disease phenotypes were also considered. We combined different types of data sets respectively, used machine learning methods (including LR, RF, and XGBoost) and different sampling methods (oversampling and SMOTE algorithm) to choose the best data set, sampling method, and model. All data sets were cross-validated five times on the model and the result was the average of the five AUC values. In the end, we found that XGBoost performed the best (AUC value was 0.88) in the data set that cell subsets combined with clinical information was balanced through oversampling. The same performances were the cell subpopulations combined with clinical information, laboratory indicators, and disease phenotypes, which was balanced through the SMOTE algorithm or oversampling, and the AUC values of XGBoost were also 0.88. The three results were the highest among all model results (see Table 4).

**Table 4:** Receiver operating characteristic curve (ROC) analysis of the different models combined with different data sets and sampling methods showed area under the curves (AUC).

**Sample modes**	**Models**	**Cell**	**Cell subset+**	**Cell subsets +**	**Cell subsets +**	**Cell subsets +**
		**subsets**	**clinical**	**laboratory**	**phenotypes**	**clinical + laboratory**
				**indicators**		**indicators phenotypes**
Raw data	Lasso	0.50	0.50	0.50	0.50	0.50
	RF	0.51	0.56	0.50	0.52	0.52
	XGBoost	0.60	0.70	0.47	0.45	0.59
Over-sampling	Lasso	0.63	0.82	0.68	0.71	0.87
	RF	0.65	0.75	0.78	0.73	0.80
	XGBoost	0.73	**0.88**	0.80	0.82	**0.88**
SMOTE	Lasso	0.65	0.77	0.64	0.80	0.84
	RF	0.78	0.81	0.83	0.86	0.80
	XGBoost	0.86	0.84	0.84	0.84	**0.88**

*The three results performing the highest among all model results are bold*.

We initially screened out the best sampling methods (oversampling and SMOTE algorithm), the best data set (cell subsets combined with clinical information and cell subsets combined with clinical information, laboratory indicators, and disease phenotypes), and the best model (XGBoost). The rebalanced data set was no longer five-fold cross-validation but a ratio of seven to three. We found that cell subsets combined with basic clinical information, laboratory indicators, and disease phenotypes, balanced through SMOTE, was shown to perform best (see Table 5). The AUC value of XGBoost was 0.922, which was much higher than the models established on the cell subpopulations data combined with the clinical information through oversampling (AUC value was 0.866) and cell subsets combined with basic clinical information, laboratory indicators, and disease phenotypes through oversampling (AUC value was 0.815). We selected the top 25 characteristics of the model.

**Table 5:** The selection of the best model.

**Sample**	**Cell subsets+**	**Cell subsets +**
**modes**	**clinical**	**clinical + laboratory**
		**indicators + phenotypes**
Oversampling	0.866	0.815
SMOTE	/	0.922

Finally, to list the characteristics related to SLE-A and SLE-NA group, there were 22 different characteristics by independent sampling with *T*-test, etc., and the range of 22 main difference characteristics was further reduced to 10 by XGBoost analysis. BMI, fatigue, depression, unstable emotions, and CD27^+^CD28^+^ Th/Treg, CD27^−^CD28^−^Th/Treg, CD45RA^−^CD27^−^ Th, and CD45RA^+^HLADR^+^ Th cells may be important characteristics of the differences (see Table 6).

**Table 6:** The selection of important characteristics.

**Mann-Whitney *U*-test or independent sample of *T*-test or chi-square test**	**XGBoost**
BMI	√
Income	
Depression	√
Sleep disorders	
Fatigue	√
Weak	
Uncomfortable in the eyes	
Less appetite	
Unstable emotions	√
γδ2T	
CD27^−^CD28^+^ Th	
CD27^+^CD28^+^ Th	√
CD27^−^CD28^−^ Th	√
CD45RA^−^CD27^−^ Th	√
CD45RA^+^HLADR^+^ Th	√
CD45RA^−^HLADR^−^ Th	
CD27^−^CD28^+^ Treg	
CD27^+^CD28^+^ Treg	√
CD27^−^CD28^−^ Treg	√
PD1^−^CD28^−^ Treg	
CD45RA^−^CD27^−^ Treg	
CD45RA^−^HLADR^−^ Treg	

## Discussions

Psychoneuroimmunology research has revealed strong associations between dysfunctions of the immune system and mental disorders (16). Several psychiatric disorders, such as schizophrenia, had been suggested to be classified as an autoimmune diseases based on the associations between the remitting-relapsing phenotype of the illness and activation-repression of immunological processes (25). Our findings showed that the incidence of SLE patients with anxiety was 22%, much higher than that of normal adults (10–14%) (26) and more likely to have symptoms of fatigue and depression. Anxiety, depression, and fatigue often co-exist as manifestations of each other (27, 28). Previous studies have shown that fatigue is strongly associated with anxiety and depression in SLE and RA patients (29–31). Increasing evidence of the role of inflammation in mental illness and the link between autoimmune diseases and mental illness is helping to expand the field of immunopsychiatry and have an impact on patient treatment and outcomes. Among inflammatory phenotypes characterizing patients with depression and anxiety, the patients have autoimmune diseases to be at high risk (4, 32, 33). In particular, SLE patients with anxiety and depression have specificity disease phenotypes that involve multiple organs (34). This supports one point at least, that in SLE patients with anxiety, the management of depression, fatigue, and anxiety may not only be a separate entity but a whole way of looking at treatment better. Similar to patients with generalized anxiety disorder (35), SLE patients with anxiety could also experience unstable emotions. In addition, we found that almost all SLE patients were women, and the average BMI of the SLE-A group was abnormal or obese. It was also traceable that obesity is an important risk factor for anxiety and depression (36), and BMI is one of the predictors of severe anxiety symptoms in women, but this result did not appear in male controls (37). Interestingly, half of the SLE-NA group had lower income than the SLE-A group, which was mainly due to the lower number of patients in the SLE-A group, and the fact that some patients did not disclose their actual income.

Statistically significant differences in 13 T cell subsets between the SLE-A and SLE-NA groups were found in our study (see Table 3). Among these immune cells, γδ2T cells are involved in the development of autoimmune diseases, including SLE (38). Our results suggested that γδ2T cells may also be involved in psychiatric disorders. Meanwhile, CD4^+^CD27^+^CD28^+^ Th and Treg cells were significantly reduced in SLE patients with anxiety. Previous studies have reported that the number of CD4^+^CD27^+^CD28^+^ cells in elderly patients with psychosis is lower than that in young patients (39). We found that the proportion of these cells might also be involved in SLE anxiety complications. Other immune components, such as CD27^−^CD28^+^, CD45RA^−^CD27^−^, and CD45RA^−^HLADR^−^Th cells, might be risk factors for psychiatric disorders associated with autoimmune diseases. Cell subsets were entirely concentrated on CD4^+^ T cells rather than CD8^+^ T cells, which was similar to the results of the previous study (14).

Our final results were BMI, fatigue, depression, unstable emotions, and the proportion of CD27^+^CD28^+^ Th/Treg, CD27^−^CD28^−^ Th/Treg, CD45RA^−^CD27^−^ Th, and CD45RA^+^HLADR^+^ Th cells (see Table 6). The overweight body shape and the negative emotions caused the extremely poor mental state, which may cause a lack of confidence and increase social fear and psychological burden. When patients are under long-term malignant psychological stress state continuously, their bodies will be in a state of systemic low-grade inflammation activation for a long time (40), which destroys the amygdala, the first unit of information processing, resulting in the destruction of the blood-brain barrier (BBB) and the enhancement of permeability (41). SLE patients with mood disorders appearing amygdala injury have also been reported previously (42). The number of CD27^+^CD28^+^ Th/Treg and CD27^−^CD28^−^ Th /Treg subsets were important characteristics in our results. We believed that CD27^−^CD28^−^ Th/Treg and CD45RA^−^CD27^−^ Th were subsets of cells containing effector memory Th/Treg cells and effector Th cells (43–46), while CD27^+^CD28^+^ Th/Treg was generally considered as naive Th/Treg cells, and CD45RA^+^HLADR^+^ Th was summarized as memory stem Th cells. These double negative subsets of cells which are at the end of T cell development were significantly increased in the SLE-A group, but double-positive subsets of cells which are at the beginning of T cell development were significantly decreased. Effector Treg cells, similar to Th cells, secrete cytokines including IL-10, IFN-γ, IL-6, IL-1β, and IL-35 (8, 9). The positive correlation of IFN-γ SLE patients with anxiety, but not with depression (12). The levels of cytokines such as IFN-γ, IL-6, IL-1α, and IL-12 in patients with generalized anxiety disorder were higher than those in the control group except for serum IL-10 (10, 11). These peripheral cytokine signals continuously stimulate the endothelial cells of the BBB, entering the human brain, and excessive or long-term active inflammatory cytokines can disrupt the expression of pro-inflammatory and anti-inflammatory phenotypes of various nerve cells, thus inducing anxiety and depression-like behavior (47). Therefore, we speculated that long-term malignant psychological stress state may lead to BBB destruction in SLE patients with anxiety, and effector memory Th/Treg cells and effector Th/Treg cells increase the secretion of more IFN-γ and IL-6 into BBB, enhancing the central inflammatory response, and thus causing anxiety.

## Conclusion

Although we used SMOTE to compensate for the imbalance of the data sets and used machine learning to further select important characteristics, it was undeniable that the results of our virtual data, randomly created based on computer algorithms, might have some deviations from the actual situation. However, the final results of this study were based on the first step, using statistical methods to analyze the original data to produce significant differences, thereby ensuring that the subsequent results have a more specific reference value for other studies. In short, our research made full use of clinical information, laboratory indicators, and disease phenotypes combined with cell subsets data to study the relationship between T cells and SLE and anxiety through machine learning. Our findings indicated that the T cell subsets closely related to SLE (CD27^+^CD28^+^ Th/Treg, CD27^−^CD28^−^ Th/Treg, CD45RA^−^CD27^−^ Th, and CD45RA^+^HLADR^+^ Th) may be involved in SLE patients with anxiety. The development indirectly supplements the results of previous studies, that is, like anxiety mice, CD4^+^ T cells also play an equally important role (14). BMI, fatigue, depression, and unstable emotions also suggest that SLE patients with anxiety have complex and multiple psychological problems, which should be considered as a whole during the subsequent treatment.

## Data Availability Statement

The raw data supporting the conclusions of this article will be made available by the authors, without undue reservation.

## Ethics Statement

The studies involving human participants were reviewed and approved by the Ethics Committee of the Affiliated Hospital of Nantong University (2017-K003). The patients/participants provided their written informed consent to participate in this study.

## Author Contributions

Z-fG, X-mZ, and CD: conception and design. X-xG, YJ, TF, and YY: analysis and interpretation of data. Z-fG: project administration. Z-fG and X-xG: drafting or revising the article critically for important intellectual content. X-xG, YJ, TF, X-mZ, TL, YY, RL, WZ, J-xG, RZ, CD, and Z-fG: final approval of the version to be submitted. TL and YY: supervision. RL, WZ, J-xG, and RZ: validation. X-xG, TL, and YY: software. All authors contributed to the article and approved the submitted version.

## Funding

This work was supported by the National Natural Science Foundation of China, Grant/Award Nos. 82071838 and 82001737, Postgraduate Research & Practice Innovation Program of Jiangsu Province, Grant/Award No. KYCX20_2842, and Science and Technology Project of Nantong City: JCZ19057.

## Conflict of Interest

The authors declare that the research was conducted in the absence of any commercial or financial relationships that could be construed as a potential conflict of interest.

## Publisher's Note

All claims expressed in this article are solely those of the authors and do not necessarily represent those of their affiliated organizations, or those of the publisher, the editors and the reviewers. Any product that may be evaluated in this article, or claim that may be made by its manufacturer, is not guaranteed or endorsed by the publisher.
