# Supplementary material for: Relevant Characteristics Analysis Using Natural Language Processing and Machine Learning Based on Phenotypes and T-Cell Subsets in Systemic Lupus Erythematosus Patients With Anxiety
Source: Front Psychiatry. 2021 Dec 10;12:793505. doi: 10.3389/fpsyt.2021.793505 (PMC8703039; doi:10.3389/fpsyt.2021.793505)
Supplement: Supplementary file 1 [file Table_1.docx]

| Laboratory indicators | SLE-A  (n=23) | SLE-NA  (n=84) | P-value |
| --- | --- | --- | --- |
| CRP ^a^ | 2.09 (1.15,3.37) | 1.83 (1.21,3.68) | 0.909 |
| C3 ^b^ | 0.65±0.17 | 0.60±0.21 | 0.362 |
| C4 ^b^ | 0.15±0.12 | 0.13±0.07 | 0.394 |
| ESR ^a^ | 11.00 (2.75,27.50) | 13.50 (7.75,21.00) | 0.308 |
| Anti-dsDNA ^a^ | 23.00 (5.50,121.00) | 34.30 (14.60,132.00) | 0.510 |
| WBC ^a^ | 4.40 (3.40,6.40) | 4.60(3.53,6.50) | 0.668 |
| NEUT ^a^ | 2.86(2.28,4.18) | 3.00(2.19,4.33) | 0.785 |
| LY ^b^ | 1.27±0.74 | 1.30±0.58 | 0.868 |
| MONO ^b^ | 0.39±0.16 | 0.40±0.19 | 0.813 |
| RBC ^b^ | 4.35±0.78 | 4.27±0.51 | 0.562 |
| Hb ^b^ | 123.17±19.30 | 123.38±18.24 | 0.962 |
| PLT ^b^ | 202.78±71.50 | 213.75±88.79 | 0.587 |
| AST ^a^ | 24.00 (20.00,32.00) | 23.00 (19.00,28.75) | 0.112 |
| ALT ^a^ | 21.00 (15.00,32.00) | 23.00 (15.00,33.50) | 0.991 |
| ALB ^a^ | 75.50 (55.75,88.00) | 68.00 (52.00,84.00) | 0.691 |
| LDH ^a^ | 210.00 (177.50,250.50) | 191.00 (161.00,257.00) | 0.417 |
| Carbamide ^a^ | 5.55 (4.38,7.60) | 4.90 (3.80,6.95) | 0.426 |
| Creatinine ^a^ | 61.00 (50.00,72.00) | 51.00 (46.00,66.75) | 0.114 |
| UA^b^ | 288.06±95.26 | 270.75±101.70 | 0.517 |

**Supplementary table 1:** Laboratory indicators characteristics of SLE patients.

^a^ Values are presented as the median (25th and 75th percentiles) and analyzed by Mann-Whitney U test,

^b^ Values are presented as the mean ± SD and analyzed by independent samples T test.

The P-value is preserved by three decimal places, and the rest is preserved by two decimal places, and P-values <0.05 are bold.

Abbreviations: CRP: c-reactive protein; C3: complement 3; C4: complement 4; ESR: erythrocyte sedimentation rate; Anti-dsDNA: anti-dsDNA antibody; WBC: white blood cell count; NEUT: neutrophil count; LY: lymphocyte count; MONO: monocyte count; RBC: red blood cell count; Hb: hemoglobin; PLT: blood platelet count; AST: aspartate transferase; ALT: alanine aminotransferase; ALB: alkaline phosphatase; LDH: lactic dehydrogenase; UA: uric acid.
