# Supplementary material for: Relevant Characteristics Analysis Using Natural Language Processing and Machine Learning Based on Phenotypes and T-Cell Subsets in Systemic Lupus Erythematosus Patients With Anxiety
Source: Front Psychiatry. 2021 Dec 10;12:793505. doi: 10.3389/fpsyt.2021.793505 (PMC8703039; doi:10.3389/fpsyt.2021.793505)
Supplement: Supplementary file 2 [file Table_2.docx]

| B-cell subsets | SLE-A  (n=23) | SLE-NA  (n=84) | P-value |
| --- | --- | --- | --- |
| ASC %PBMC^a^ | 0.20 (0.10,0.58) | 0.26 (0.13,0.49) | 0.753 |
| B %PBMC^a^ | 4.60 (3.37,7.39) | 6.09 (3.13,11.35) | 0.181 |
| ASC %CD19^a^ | 3.50 (1.95,9.23) | 3.79 (1.75,7.41) | 0.906 |
| IgA^+^ %ASC^b^ | 31.09±21.43 | 29.87±21.52 | 0.809 |
| IgAM^-^ %ASC^a^ | 33.70 (26.30,69.60) | 39.95 (27.38,62.05) | 0.988 |
| IgM^+^ %ASC^a^ | 6.38 (0.96,18.10) | 10.10 (1.01,18.55) | 0.648 |
| CD138^+^ %ASC^a^ | 10.00 (4.35,15.10) | 11.05 (7.03,18.55) | 0.334 |
| Bn %B^b^ | 33.97±16.74 | 36.45±18.61 | 0.565 |
| Bim %B^a^ | 6.20 (0.58,15.10) | 6.85 (2.31,12.83) | 0.622 |
| IgM^+^ %B^a^ | 4.90 (2.73,9.31) | 4.09 (2.37,7.19) | 0.495 |
| IgM^+^D^-^ %B^a^ | 0.62 (0.10,0.94) | 0.38 (0.10,1.13) | 0.850 |
| SwBm %B^a^ | 30.60 (17.50,51.70) | 25.30 (14.65,41.70) | 0.273 |
| IgA^+^ %SwBm^a^ | 28.60 (12.90,76.90) | 33.45 (16.48,72.30) | 0.844 |
| IgA^-^ %SwBm^a^ | 64.00 (14.60,80.20) | 58.85 (22.65,79.40) | 0.744 |
| AtM %B^a^ | 6.18 (3.56,14.50) | 5.08 (2.74,9.47) | 0.369 |

**Supplementary table 2:** The abundance of 15 B-cell subsets of immune cells in SLE-A and SLE-NA groups.

^a^ Values are presented as the median (25th and 75th percentiles) and analyzed by Mann-Whitney U test,

^b^ Values are presented as the mean ± SD and analyzed by independent samples T test.

The P-value is preserved by three decimal places, and the rest is preserved by two decimal places, and P-values <0.05 are bold.

Abbreviations: PBMC: Peripheral Blood Mononuclear Cell; Bn: naïve immature B cells; Btr: transitional B cells; SwBm: switched memory B cell; ASC: plasma B cells; AtM: atypical memory B cells.
