# Supplementary material for: Relevant Characteristics Analysis Using Natural Language Processing and Machine Learning Based on Phenotypes and T-Cell Subsets in Systemic Lupus Erythematosus Patients With Anxiety
Source: Front Psychiatry. 2021 Dec 10;12:793505. doi: 10.3389/fpsyt.2021.793505 (PMC8703039; doi:10.3389/fpsyt.2021.793505)
Supplement: Supplementary file 3 [file Table_3.docx]

| T-cell subsets | SLE-A  (n=23) | SLE-NA  (n=84) | P-value |
| --- | --- | --- | --- |
| γδ1T^a^ | 0.89 (0.36,4.31) | 1.07 (0.36,1.90) | 0.679 |
| γδ2T^a^ | 1.40 (0.98,2.76) | 0.91 (0.33,1.86) | **0.029** |
| γδ1^-^2^-^ T^a^ | 0.52 (0.30,0.95) | 0.45 (0.21,0.83) | 0.476 |
| MAIT^a^ | 0.33 (0.10,0.81) | 0.18 (0.05,0.63) | 0.441 |
| iNKT^a^ | 0.10 (0.02,0.16) | 0.10 (0.05,0.20) | 0.527 |
| αβT^a^ | 89.80 (84.70,92.10) | 89.70 (85.40,92.60) | 0.832 |
| CD4^+^ T^b^ | 48.25±13.4 | 47.38±12.02 | 0.765 |
| CD8^+^ T^b^ | 47.98±13.78 | 47.05±13.95 | 0.776 |
| DPT^a^ | 0.18 (0.12,0.29) | 0.17(0.08,0.37) | 0.611 |
| DNT^b^ | 1.34±1.47 | 1.19±0.77 | 0.496 |
| Th^a^ | 96.40 (94.60,97.40) | 96.50 (94.45,98.08) | 0.823 |
| Treg^a^ | 2.50 (1.70,3.91) | 2.30 (1.20,4.41) | 0.413 |
| Tn %Th^b^ | 28.04±13.54 | 32.61±16.70 | 0.232 |
| TCM %Th^b^ | 48.80±20.37 | 49.15±18.19 | 0.936 |
| TEM %Th^a^ | 19.50 (3.32,34.10) | 10.28 (4.01,19.60) | 0.113 |
| TEMRA %Th^a^ | 0.66 (0.08,1.87) | 0.32 (0.09,1.05) | 0.450 |
| Act %Th^a^ | 3.42 (0.93,6.79) | 2.94 (1.33,5.50) | 0.527 |
| Tn %Treg^a^ | 5.10 (2.47,11.30) | 10.30 (3.48,20.30) | 0.104 |
| TCM %Treg^b^ | 68.08±20.82 | 64.32±21.55 | 0.457 |
| TEM %Treg^b^ | 21.63±17.93 | 19.38±15.40 | 0.551 |
| TEMRA %Treg^a^ | 0.63 (0.00,1.38) | 0.27 (0.00,1.012) | 0.295 |
| Act %Treg^a^ | 10.90(4.50,20.30) | 8.05 (2.74,15.35) | 0.326 |
| Tn %CD8^b^ | 33.46±21.49 | 38.73±24.09 | 0.344 |
| TCM %CD8^a^ | 6.08 (3.25,9.52) | 4.64 (2.01,7.69) | 0.285 |
| TEM %CD8^b^ | 34.53±18.27 | 35.18±20.35 | 0.891 |
| TEMRA %CD8^b^ | 22.75±14.18 | 19.73±15.12 | 0.391 |
| Act %CD8^a^ | 3.63 (1.27,6.25) | 2.01(1.05,5.23) | 0.196 |
| CD27^-^CD28^+^ Th^a^ | 13.90 (9.75,19.60) | 9.93 (6.45,14.30) | **0.026** |
| CD27^+^CD28^+^ Th^a^ | 70.60 (59.80,80.30) | 84.30 (74.80,90.48) | **0.001** |
| CD27^+^CD28^-^ Th^a^ | 0.04 (0.00,0.12) | 0.04 (0.01,0.13) | 0.942 |
| CD27^-^CD28^-^ Th^a^ | 15.40 (0.36,23.60) | 3.67 (0.48,10.70) | **0.039** |
| PD1^-^CD28^+^ Th^a^ | 67.60 (59.60,83.30) | 75.90 (45.53,87.88) | 0.567 |
| PD1^+^CD28^+^ Th^a^ | 14.10 (6.94,20.80) | 14.70 (6.28,35.53) | 0.488 |
| PD1^+^CD28^-^ Th^a^ | 1.31 (0.12,5.03) | 0.83 (0.06,3.41) | 0.430 |
| PD1^-^CD28^-^ Th^a^ | 3.99 (0.18,17.8) | 1.27 (0.02,5.82) | 0.056 |
| CD45RA^-^CD27^+^ Th^b^ | 39.61±16.95 | 45.51±14.13 | 0.092 |
| CD45RA^+^CD27^+^ Th^b^ | 28.17±13.07 | 34.01±17.09 | 0.132 |
| CD45RA^+^CD27^-^ Th^a^ | 0.76 (0.20,2.43) | 0.44 (0.24,1.21) | 0.168 |
| CD45RA^-^CD27^-^ Th^b^ | 28.77±12.45 | 18.70±14.30 | **0.003** |
| CD45RA^-^HLADR^+^ Th^a^ | 12.10 (4.10,54.40) | 31.10 (4.56,61.25) | 0.387 |
| CD45RA^+^HLADR^+^ Th^a^ | 0.35 (0.11,16.00) | 11.25 (0.29,34.50) | **0.034** |
| CD45RA^+^HLADR^-^ Th^a^ | 20.70 (4.40,32.40) | 4.84 (0.40,37.33) | 0.225 |
| CD45RA^-^HLADR^-^ Th^a^ | 50.20 (4.62,65.60) | 6.35 (1.21,55.38) | **0.035** |
| CD27^-^CD28^+^ Treg^a^ | 8.40 (4.02,12.80) | 4.69 (2.47,8.78) | **0.044** |
| CD27^+^CD28^+^ Treg^a^ | 84.50 (78.30,92.00) | 93.10 (86.38,96.78) | **0.005** |
| CD27^+^CD28^-^ Treg^a^ | 0.00 (0.00,0.20) | 0.00 (0.00,0.06) | 0.177 |
| CD27^-^CD28^-^ Treg^a^ | 3.54 (0.00,9.64) | 0.45(0.00,2.40) | **0.032** |
| PD1^-^CD28^+^ Treg^a^ | 74.20 (49.50,84.50) | 69.40 (34.90,85.00) | 0.785 |
| PD1^+^CD28^+^ Treg^a^ | 17.70 (12.20,27.30) | 25.60 (12.03,65.10) | 0.214 |
| PD1^+^CD28^-^ Treg^a^ | 0.16 (0.00,1.84) | 0.00 (0.00,1.39) | 0.440 |
| PD1^-^CD28^-^ Treg^a^ | 2.85 (0.00,8.21) | 0.00 (0.00,1.685) | **0.012** |
| CD45RA^-^CD27^+^ Treg^a^ | 77.30 (66.30,85.30) | 79.70 (62.83,87.90) | 0.606 |
| CD45RA^+^CD27^+^ Treg^a^ | 5.95 (1.84,12.30) | 9.75(3.70,22.88) | 0.200 |
| CD45RA^+^CD27^-^ Treg^a^ | 0.07 (0.00,1.08) | 0.00 (0.00,0.42) | 0.219 |
| CD45RA^-^CD27^-^ Treg^a^ | 12.80 (7.61,17.90) | 7.01 (3.17,13.25) | **0.009** |
| CD45RA^-^HLADR^+^ Treg^b^ | 49.47±23.61 | 55.78±29.85 | 0.351 |
| CD45RA^+^HLADR^+^ Treg^a^ | 0.62 (0.16,5.00) | 1.81(0.13,13.40) | 0.309 |
| CD45RA^+^HLADR^-^ Treg^a^ | 3.45 (1.47,7.25) | 1.79 (0.00, 6.90) | 0.132 |
| CD45RA^-^HLADR^-^ Treg^a^ | 42.60 (9.26,58.70) | 11.85 (1.19,55.63) | **0.032** |
| CD27^-^CD28^+^ CD8^a^ | 3.12 (2.06,5.16) | 2.33 (1.16,4.15) | 0.09 |
| CD27^+^CD28^+^ CD8^b^ | 41.79±20.41 | 47.79±22.42 | 0.250 |
| CD27^+^CD28^-^ CD8^b^ | 8.84±6.15 | 8.05±7.15 | 0.629 |
| CD27^-^CD28^-^ CD8^b^ | 43.52±22.19 | 39.61±22.66 | 0.463 |
| PD1^-^CD28^+^ CD8^b^ | 41.61±25.72 | 36.73±28.88 | 0.464 |
| PD1^+^CD28^+^ CD8^a^ | 4.04(1.87,7.09) | 3.94 (2.13,21.75) | 0.476 |
| PD1^+^CD28^-^ CD8^a^ | 2.73 (1.05,13.30) | 4.21(1.11,21.50) | 0.417 |
| PD1^-^CD28^-^ CD8^b^ | 38.41±23.91 | 33.18±27.41 | 0.407 |
| CD45RA^-^CD27^+^ CD8^b^ | 17.21±9.17 | 13.89±8.63 | 0.109 |
| CD45RA^+^CD27^+^ CD8^b^ | 36.40±20.03 | 40.98±22.51 | 0.379 |
| CD45RA^+^CD27^-^ CD8^a^ | 16.30 (10.10,27.80) | 15.75 (7.17,24.85) | 0.419 |
| CD45RA^-^CD27^-^ CD8^b^ | 27.49±15.73 | 27.04±16.72 | 0.909 |
| CD45RA^-^HLADR^+^ CD8^a^ | 4.21 (1.28,19.20) | 5.59 (1.96,13.35) | 0.762 |
| CD45RA^+^HLADR^+^ CD8^a^ | 2.27 (0.42,5.37) | 1.88 (0.58,5.62) | 0.799 |
| CD45RA^+^HLADR^-^ CD8^b^ | 53.27±19.93 | 54.64±19.22 | 0.765 |
| CD45RA^-^HLADR^-^ CD8^b^ | 32.82±16.15 | 31.95±16.25 | 0.821 |

**Supplementary table 3:** The abundance of 75 T-cell subsets of immune cells in SLE-A and SLE-NA groups.

^a^ Values are presented as the median (25th and 75th percentiles) and analyzed by Mann-Whitney U test,

^b^ Values are presented as the mean ± SD and analyzed by independent samples T test.

The P-value is preserved by three decimal places, and the rest is preserved by two decimal places, and P-values <0.05 are bold.

Abbreviations: iNKT-cells: invariant natural killer T-cells; MAIT: mucosal associated invariant T cell; DPT: CD4^+^CD8^+^ T cells; DNT: CD4^-^CD8^-^ T cells; Th: helper T cells; Treg: regulatory T cells; Tn: naïve T cells; TCM: central memory T cells; TEM: effective memory T cells; TEMRA: effector memory RA T cells; Act: active T cells.
