# Supplementary material for: Relevant Characteristics Analysis Using Natural Language Processing and Machine Learning Based on Phenotypes and T-Cell Subsets in Systemic Lupus Erythematosus Patients With Anxiety
Source: Front Psychiatry. 2021 Dec 10;12:793505. doi: 10.3389/fpsyt.2021.793505 (PMC8703039; doi:10.3389/fpsyt.2021.793505)
Supplement: Supplementary file 4 [file Table_4.docx]

| T  markers | Fluorochrome | Company | B markers | Fluorochrome | Company |
| --- | --- | --- | --- | --- | --- |
| CD3 | BUV395 | BD | CD3 | PECy5.5 | Biolegend |
| CD4 | PECy5.5 | eBioscience | CD11c | BV650 | eBioscience |
| CD8 | A700 | Biolegend | CD19 | BV785 | Biolegend |
| CD25 | APC | Biolegend | CD20 | BV711 | Biolegend |
| CD27 | BV785 | Biolegend | CD21 | PECy5 | Biolegend |
| CD28 | PE | Biolegend | CD24 | PC594 | Biolegend |
| CD38 | FITC | eBioscience | CD27 | PE | BD |
| CD45RA | BV711 | Biolegend | CD38 | PECy7 | BD |
| CD161 | BV605 | Biolegend | CD45 | BV570 | Miltenyi |
| CD127 | BV650 | Biolegend | CD138 | APC | Biolegend |
| TCRαβ | PECy5 | Biolegend | IgD | BV510 | Biolegend |
| TCRVδ_1_ | PerCP-V700 | Miltenyi | IgM | A700 | Biolegend |
| TCRVδ_2_ | APC | Biolegend | IgA | FITC | BD |
| TCRVα7.2 | PerCP-V700 | Miltenyi | IgG | Biotin | Biolegend |
| TCRVα24 | BV510 | Biolegend |  |  |  |
| CCR7 | PE-Dazzle | Biolegend |  |  |  |
| HLADR | PECy7 | Biolegend |  |  |  |
| PD1 | BV421 | Biolegend |  |  |  |
| Sav | APC-eF780 | Biolegend |  |  |  |

**Supplementary table 4:** Antibodies used for cell selection in flow cytometry.

Antibodies used for immunophenotyping by flow cytometry, target marker, conjugated fluorochrome and company displayed.
