# Supplementary material for: Relevant Characteristics Analysis Using Natural Language Processing and Machine Learning Based on Phenotypes and T-Cell Subsets in Systemic Lupus Erythematosus Patients With Anxiety
Source: Front Psychiatry. 2021 Dec 10;12:793505. doi: 10.3389/fpsyt.2021.793505 (PMC8703039; doi:10.3389/fpsyt.2021.793505)
Supplement: Supplementary file 5 [file Table_5.docx]

| R packages | Versions | Functions |
| --- | --- | --- |
| DMwR | v0.4.1 | SMOTE |
| ROSE | v0.0.4 | Oversampling |
| glmnet | v4.1.2 | Lasso Regression |
| varSelRF | v0.7.8 | Random Forest |
| xgboost | v1.4.1.1 | XGBoost |
| jiebaR | v0.11 | Natural Language Processing |
| ROCR | v1.0.11 | ROC |
| pROC | v1.18.0 | ROC |

**Supplementary table 5:** The main R packages used in the analysis process.

Abbreviations: SMOTE: Syntic priority oversampling technology; ROC: Receiver operator characteristic.
